# Pulsed Electrical Stimulation Affects Osteoblast Adhesion and Calcium Ion Signaling

**DOI:** 10.3390/cells11172650

**Published:** 2022-08-25

**Authors:** Susanne Staehlke, Meike Bielfeldt, Julius Zimmermann, Martina Gruening, Ingo Barke, Thomas Freitag, Sylvia Speller, Ursula Van Rienen, Barbara Nebe

**Affiliations:** 1 Department of Cell Biology, Rostock University Medical Center, 18057 Rostock, Germany; 2 Department of Computer Science and Electrical Engineering, Institute of General Electrical Engineering, University of Rostock, 18059 Rostock, Germany; 3 Physics of Surfaces and Interfaces, Institute of Physics, University of Rostock, 18059 Rostock, Germany; 4 Department Life, Light & Matter, Interdisciplinary Faculty, University of Rostock, 18059 Rostock, Germany; 5 Department Aging of Individuals and Society, Interdisciplinary Faculty, University of Rostock, 18059 Rostock, Germany

**Keywords:** electrical stimulation, electric field strength, field simulation, AC-stimulated liquid, osteoblasts adhesion, spreading, calcium ions, reactive oxygen species, confocal microscopy, scanning electron microscopy

## Abstract

An extensive research field in regenerative medicine is electrical stimulation (ES) and its impact on tissue and cells. The mechanism of action of ES, particularly the role of electrical parameters like intensity, frequency, and duration of the electric field, is not yet fully understood. Human MG-63 osteoblasts were electrically stimulated for 10 min with a commercially available multi-channel system (IonOptix). We generated alternating current (AC) electrical fields with a voltage of 1 or 5 V and frequencies of 7.9 or 20 Hz, respectively. To exclude liquid-mediated effects, we characterized the AC-stimulated culture medium. AC stimulation did not change the medium’s pH, temperature, and oxygen content. The H_2_O_2_ level was comparable with the unstimulated samples except at 5 V_7.9 Hz, where a significant increase in H_2_O_2_ was found within the first 30 min. Pulsed electrical stimulation was beneficial for the process of attachment and initial adhesion of suspended osteoblasts. At the same time, the intracellular Ca^2+^ level was enhanced and highest for 20 Hz stimulated cells with 1 and 5 V, respectively. In addition, increased Ca^2+^ mobilization after an additional trigger (ATP) was detected at these parameters. New knowledge was provided on why electrical stimulation contributes to cell activation in bone tissue regeneration.

## 1. Introduction

A widespread research field in regenerative medicine is electrical stimulation (ES), and its impact on tissue and cells, such as bone [1]. In 1957 the piezoelectric properties of bone were described [2]. As the bone healing process takes place under the mechanical strain of the bone [3], an electric field is generated in the bone in vitro and in vivo [4]. Externally applied electric fields were shown to contribute to bone deposition and osteoblast differentiation and proliferation [5]. The living cell has a membrane potential that indicates the electrical potential difference between the intracellular and extracellular space, which is generated by the transport of ions through ion channels and ion transporters [6]. The membrane potential is always negative because of the higher negative ion concentration inside the cell [7,8]. Primary osteoblasts revealed a membrane potential of −60 mV [9], MG-63 osteoblasts demonstrated a negatively charged cell surface of −15.6 mV [10]. Due to the appearance of ions and polar or charged molecules, cells can build up electrical fields or can respond to them [11,12]. If injuries appear on the bone, a wound potential, which should lead precursor cells to differentiation, will be generated [13]. A possible approach for the therapy is through electrical stimulation to accelerate bone healing. In some publications, it is described that ES can manipulate different cell processes, for example, cell adhesion, proliferation, apoptosis, cell migration, and differentiation [8,14]. Widespread methods of the ES for bone tissue are invasive methods like direct contact stimulation [14], in vitro, the electrically conductive electrodes are placed directly in the culture medium. The applied current streams from the anode to the cathode [15]. The modification of the cell membrane potential through ES can activate different intracellular pathways. External electrical fields can generate an intracellular calcium ion (Ca^2+^) flow and consequently activate different pathways [16], such as wnt [17]. Ca^2+^ dynamics were identified as a sensitive parameter for cell physiological processes. Furthermore, the ES can stimulate the production of reactive oxygen species (ROS), which can control different cellular processes like proliferation and modulated signaling pathways [18,19]. When the equilibrium is destroyed, and the concentration of ROS is relatively high, cells suffer under oxidative stress. Moreover, Ca^2+^ signaling was shown to contribute to ROS generation through calmodulin [20], as well as ROS contributing to increased intracellular Ca^2+^ concentrations by altering calcium channels [21].

It was described that the electric field could modulate cell functions in order to accelerate wound healing and bone regeneration [5,12,14]. In contrast to previous studies, we are focusing on the influence of a short alternating current (AC)-stimulation time on settling cells via a commercial device (IonOptix) with the following parameters: rectangular waveform, voltages of ±1 or ±5 V, frequency of 7.9 Hz with pulse duration of 10 ms, or 20 Hz with pulse duration of 3.6 ms. In vitro, cell processes such as intracellular Ca^2+^ level and mobilization, adhesion, and morphology of human osteoblasts within 24 h were studied to understand the effect of ES on osteoblast behavior. In addition, we wanted to shed light on the question of whether ES affects the cells via liquids, i.e., AC-pre-conditioned medium. For these cell experiments, solely the cell culture medium was stimulated in the IonOptix chamber and analyzed for pH, temperature, oxygen, and hydrogen peroxide content.

## 2. Materials and Methods

### 2.1. Electrical Stimulation (ES) Chamber C-Pace EM 100

**Figure 1:**
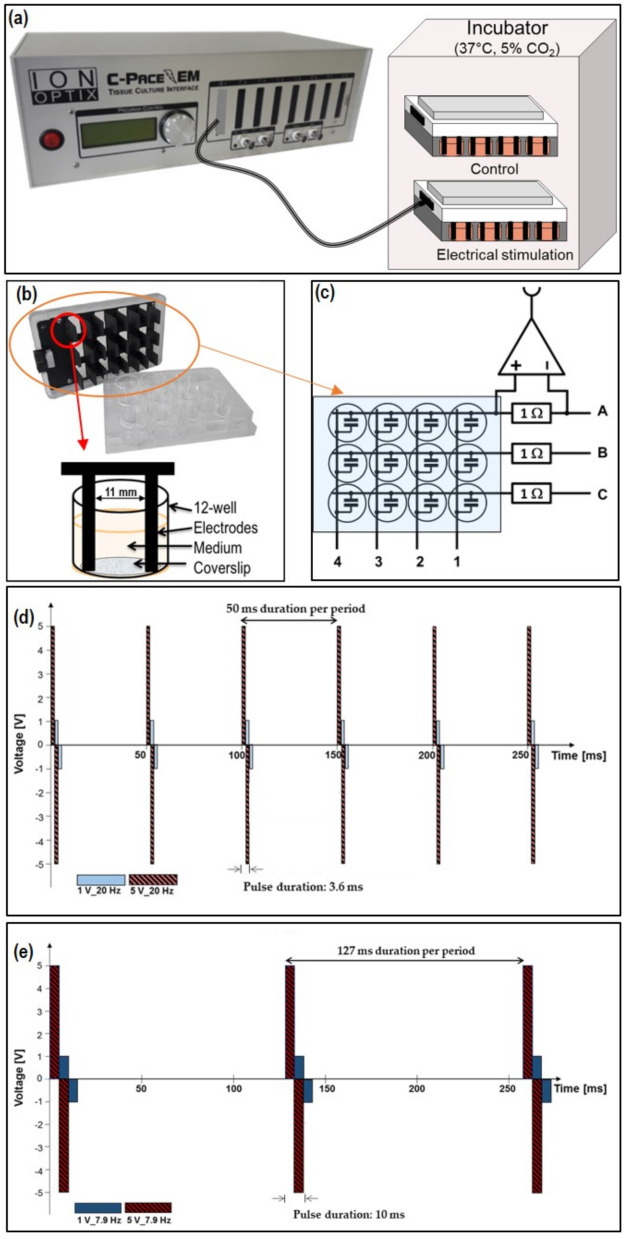
Scheme of the AC electrical stimulation system. (**a**) IonOptix stimulation chamber C-Pace EM 100—multi-channel stimulator, (**b**) graphite electrodes for a 12-well plate format reaching the bottom, and (**c**) simplified scheme of the 12 well C-Dish. For the current measurement, each column of the 12-well plate (background) is connected to the IonOptix controller via a shunt resistor. The corresponding voltage drop is amplified by an instrumentation amplifier which can be connected to any of the columns (not shown). In (**d**) illustration of rectangular electronic bipolar pulses of the AC stimulation with 20 Hz, and (**e**) 7.9 Hz. The pulse duration covers both positive and negative half pulses.

For electrical stimulation (ES), we used a multi-channel electrical stimulator, a voltage generator, and a 12-well C (culture)-Dish (IonOptix, Milton, MA, USA) (Figure 1a,c).

IonOptix devices were used for the electrical and mechanical in vitro stimulation of cells, e.g., stem cells, cardiogenic and osteoblasts, or for tissues [12,14].

The stimulation chamber C-Pace EM 100 is a multi-channel stimulator that enables the alternating current (AC)-stimulation of cells in an incubator. The electrodes are designed for a 12-well plate (Greiner bio-one, Frickenhausen, Germany), and reach down to the bottom of the wells. This way, all cells can be electrically stimulated. The electrodes consist of carbon in the graphite modification and are shaped in a plate format (capacitor). The advantages of graphite are its excellent electrical conductivity and the high resistance against corrosion. The protein adsorption on the surface is problematic as it affects the electrical field´s homogeneity. Therefore, a regular cleaning after use is necessary [22]. The electrodes can be sterilized by treatment with ethanol and ultraviolet light (UV). The space between both plate electrodes amounts to 1.1 cm, and the wetted area in a well of the 12-well plate totals 0.42 cm^2^ when 1 mL fluid is in the well (Figure 1b). To always establish identical conditions of the experiments, coverslips (Menzel™ Microscope Coverslips, Ø 18 mm, Thermo Fisher Scientific, Waltham, MA, USA) were placed on each bottom of the 12-well plate.

This ES-chamber generates rectangular electronic biphasic pulses for electrical stimulation. The parameters can be varied: the applied voltage up to ±40 V, the frequencies from 0.01 to 99 Hz, and the pulse duration from 0.4 to 10 ms [12,14]. Every well is gated in a definite sequence.

In general, we used 1 and 5 V for the experiments, and a frequency of 20 Hz (3.6 ms double duration time, ± V) (1 V_20 Hz, 5 V_20 Hz) (Figure 1d) or 7.9 Hz (10 ms double duration time, ± V) (1 V_7.9 Hz, 5 V_7.9 Hz) (Figure 1e). After seeding, the cells in the 12-well plates were directly stimulated for 10 min. For the controls we worked with the C-Dish electrodes but without electrical stimulation (system still switched off) (Control).

### 2.2. Calculation and Simulation of ES

The applied electric field strength was evaluated by employing numerical simulations. Under the assumption that the dielectric parameters of the cell culture medium are not frequency-dependent, the electrostatic Laplace equation is solved [23]. This equation reads

Δϕ=0

This yields the electric potential (ϕ). Subsequently, the electric field can be computed by taking the gradient of the potential. As the solution depends linearly on the voltage difference between the two plates, we evaluated the electric field at a voltage difference of 1 V. In the case of a different voltage difference, the solution can be linearly scaled. We used the finite element method for our computations, implemented in the open-source package EMStimTools (https://github.com/j-zimmermann/EMStimTools/releases/tag/v0.1.4.dev0; accessed on 27 July 2022) [24]. The geometry of a single well was used and represented by a 2D model. The 2D representation corresponds to a slice through the geometry at a certain height and the top view, respectively.

### 2.3. Current Measurement

The electrodes of the 12-well plate are arranged in a 4 × 3 matrix such that individual wells can be addressed sequentially by applying the stimulation voltage to the respective row and column, see Figure 1c. Current measurement was accomplished via a shunt resistor (R = 1 Ω) inserted into each column, enabling the sequential measurement of all four wells in the same column. An instrumentation amplifier with variable gain and high bandwidth (up to 500 kHz) was used to precisely convert the differential shunt voltage drop to a single-ended output voltage. The stated currents corresponded to the peak values of the time-dependent signal.

### 2.4. Characterization of AC-Stimulated Liquid

To eliminate the possibility that the electric field influences the medium alone and thus influences the cell physiology, the medium parameters temperature, pH, oxygen, and hydrogen peroxide (H_2_O_2_) content were analyzed. For temperature, pH, and oxygen, fresh Dulbecco’s Modified Eagle’s Medium (DMEM; Thermo Fisher Scientific, Waltham, MA, USA) with 10% fetal calf serum (FCS; Biochrom FCS Superior, Merck KGaA, Darmstadt, Germany) and 1% gentamicin (Ratiopharm GmbH, Ulm, Germany) was exposed to ES for 10 min in a 12-well plate (1 mL per well). These parameters were measured in the middle of the liquid volume in the incubator at 37 °C, and 5% CO_2_ in triplicate: (i) temperature with the device Mini-K (Dostmann, with temperature gauger for Type K probes by testo), (ii) pH with a pH meter (Sentron SI series pH meter, SI400-010; Sentron Europe BV, Leek, The Netherlands), and (iii) oxygen with a needle-shaped optical oxygen microsensor (Oxygen micro-optode, type PSt1; Presens) connected to the Microx TX3 (Presens).

The H_2_O_2_ concentration was determined using the Fluorimetric Hydrogen Peroxidase Assay kit (Sigma Aldrich, Merck KGaA, Darmstadt, Germany). For H_2_O_2_ measurement, DMEM (without pyruvate; Life technologies, Thermo Fisher Scientific) with 10% FCS was 10 min AC-stimulated. H_2_O_2_ content was analyzed at time points 0, 0.5, 4, and 24 h, and the probes were kept at 37 °C.

For all Controls, the electrodes were placed in the medium, and the power switch was not returned on (no ES).

### 2.5. Cell Culture

The cell biological investigations were performed with human osteoblast-like cells MG-63 (passage number 5 to 30, ATCC^®^ CRL-1427™, Manassas, VA, USA) [25]. The MG-63s represent a well-established osteoblast model due to the similar characteristics in terms of cell spreading, adhesion, and signaling properties as primary human osteoblasts [25,26,27]. The MG-63s were cultivated in complete DMEM (with pyruvate, Thermo Fisher; 10% FCS; see Section 2.4.) at 37 °C in an incubator with 5% CO_2_. To obtain suspended cells for the experiments, cells were trypsinated (0.05% trypsin-EDTA, ethylenediaminetetraacetic acid; PAA Laboratories, Pasching, Australia), collected in a complete medium and centrifuged (13,000× *g*, 4 °C, 5 min; Eppendorf 5702R; Hamburg, Germany).

### 2.6. AC-Activated Medium and Online Monitoring of Cell Adhesion

To solve the question of whether the electrostimulation effects on cells are mediated via the liquid, the medium alone was AC-activated. For long-time cell adhesion and spreading, the xCELLigence RTCA (Real-time Cell Analysis) S16 instrument (ACEA Biosciences, San Diego, CA, USA) was used [28]. The impedance-based assay determines the ion environment at the microelectrode/solution interface. The system displays the impedance in an arbitrary unit—Cell Index, which is determined by the cell-microelectrode impedance minus the baseline-impedance (medium without cells).

The E-plate VIEW 16 (ACEA Biosciences, similar to 96-well format) was pre-incubated in an incubator at 37 °C for 2 h before the experiments started [29]. The complete DMEM was first exposed to AC electric fields for 10 min. Then, MG-63s (10,000 cells/well) were seeded into the E-plate in 200 µL of the appropriate AC-stimulated medium. The impedance change caused by cell adhesion, and spreading was measured continuously at 37 °C and 5% CO_2_ up to 24 h every 8 min. Data were analyzed using RTCA Data Analysis Software 1.0. The software calculated the slope of the cell adhesion over time, the Cell Index value after 24 h (Max Cell Index), and the adhesion rate (Cell Index at the time point when 50% of the cells adhere). MG-63s long-time adhesion was presented for five independent experiments.

### 2.7. AC stimulation of Cells and Initial Adhesion

The question arose whether cells in suspension are already activated by AC and thus their adhesion behavior is altered. To hold the cells (1 × 10^6^) in suspension, MG-63s were shaken (ThermoMixer C; Eppendorf AG, Hamburg, Germany) in a complete medium at 300 rpm at 37 °C. For the initial adhesion approach, 40 µL of suspended MG-63s was seeded into one well (12-well plate) containing a coverslip, electrodes were placed, and ES was started. After 10 min, 300 µL of the non-adherent MG-63s in the supernatant were counted by the flow cytometer FACSCalibur (BD Biosciences, San Jose, CA, USA) using CellQuest Pro 4.0.1 (BD) software for data acquisition. The number of adherent osteoblasts was calculated in percent relative to the cell number at 0 min (suspended cells without adhesion on glasses). A new seeding of MG-63s with different ES parameters was performed every 10 min within a series of approaches with the same cell vial. Initial adhesion assays were performed seven times in independent experiments.

### 2.8. Cell Morphology and Spreading

At 0.5, 4, and 24 h after electrical stimulation (ES), the medium was removed, and the cells on coverslips in the wells were washed with PBS and fixed with 2.5% glutardialdehyde (Merck KGaA, Darmstadt, Germany) solution in PBS. The samples were stored until further processing at 4 °C. The fixed samples were washed with 0.1 M sodium–phosphate buffer (Merck KGaA), then they were dehydrated in an increasing ethanol solution and dried with the critical point dryer Emitech K850 (Quorum Technologies Ltd., East Sussex, UK) [29]. The dried samples were affixed on sample holders with the aid of conducting carbon tape and sputtered with a thin gold layer (10–15 nm; EM SCD 004, BAL-TEC, Balzers, Liechtenstein). The samples were analyzed by scanning electron microscopy (FE-SEM, MERLIN^®^ VP Compact, Carl Zeiss, Oberkochen, Germany) with the following instrumentation: HE-SE2 detector, acceleration voltage 5 kV, and working distance 5–7 mm.

The FE-SEM images were processed with the program ImageJ (National Institutes of Health, Bethesda, USA) to calculate the cell area. Therefore, 40 cells per treatment were measured.

### 2.9. Calcium Mobilization and AC stimulation

The membrane-permeable calcium indicator fluo-3/AM (Life Technologies Corporation, Eugene, OR, USA) was used [30], to detect if AC mobilizes calcium ions (Ca^2+^) in the cytosol. Fluo-3/AM consists of molecules with two free binding sites that display an increase in fluorescence in the presence of free Ca^2+^.

**Figure 2:**
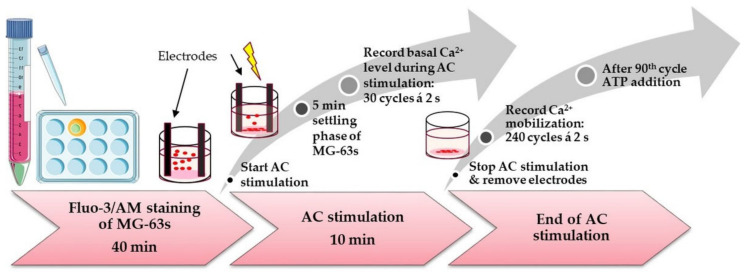
Scheme of experimental approaches of AC stimulation and calcium ions (Ca^2+^)-imaging in confocal microscopy. At first, we stained the suspended MG-63s with fluo3/AM for 40 min. Stained cells were then seeded into the 12-well plate under the microscope (LSM780, Carl Zeiss). Next, the electrodes were placed, and the electrical stimulation started. After a short settling phase of cells, a time series was done to measure the basal Ca^2+^ activity during ES (60 s). After 10 min ES, the electrodes were removed, and a second time series was recorded immediately to analyze the Ca^2+^ release and reaction after ATP addition (480 s; 240 cycles each 2 s, after 180 s ATP addition). (Parts of the figure were drawn by using pictures from Servier Medical Art. Servier Medical Art by Servier is licensed under a Creative Commons Attribution 3.0 Unported License (https://creativecommons.org/licenses/by/3.0/, accessed on 21 July 2022)).

MG-63s in a near confluent state (70–80%) were washed with PBS, trypsinized, and centrifuged (Centrifuge 5810 R, Eppendorf AG). Next, 2 × 10^6^ cells/2 mL were then resuspended in complete medium DMEM with shaking at 37 °C (ThermoMixer C). For every experiment, 2.5 × 10^5^ cells from this pool were rewashed and centrifuged, and the cell sediment was stained with fluo-3/AM (5 μM) in slightly hypotonic 4-(2-hydroxyethyl)-1-piperazineethanesulfonic acid (HEPES) buffer [31], with shaking at 37 °C. After 40 min, cells were centrifuged and resuspended in 250 µL of isotonic HEPES (formulation described in [32]). Next, 750 µL of isotonic HEPES was added to a 12-well plate (glass bottom, Cellvis, Mountain View, CA, USA), the stained suspended cells were added, and stimulation was started immediately. After 10 min, AC stimulation was stopped, and electrodes were removed. Both, during stimulation and after the 10 min AC stimulation, time series visualized the calcium signal of MG-63s using an LSM780 inverted microscope (Carl Zeiss, Jena, Germany, argon ion laser at 488 nm) with a C Apochromat 40× water immersion objective (Carl Zeiss, 1.20 W Korr M27). The acquisition of the Ca^2+^ signal was made using Zen2011 (black edition, Carl Zeiss, Jenna, Germany). The first time series during stimulation included 150 cycles every 2 s, whereby the Ca^2+^ signal was recorded upon stimulation after a 5 min settling phase of the cells. After the 10 min AC stimulation, the electrodes were removed, and the second time series was started with 240 cycles at 2 s intervals, whereby after the 90th cycle, 10 mM adenosine-50-triphosphate (ATP; SERVA Electrophoresis GmbH, Heidelberg, Germany; dissolved in PBS) was added. The mean fluorescence intensity (MFI) analysis was evaluated with Zen2012 software (blue edition, version 2.0.0.0, Carl Zeiss). The mean ROI for 10 defined areas of individual cells for each time point (240 cycles = 240 time points) was determined. In preliminary experiments, the cell areas went out of focus after approximately 60 s AC stimulation (not in Controls), so only the first 30 cycles could be evaluated. At least 6 independent experiments were analyzed to see the influence of AC stimulation on calcium–ion dynamics (Figure 2). In all experiments, the Ca^2+^ signal was recorded under the same settings (gain, digital offset) and in a pinhole with a maximum airy unit (15 AU, 13.5 μm cross-section) [32,33].

### 2.10. Cellular Reactive Oxygen Species (ROS)

Intracellular reactive oxygen species (ROS) were detected via dichloro-dihydro-fluorescein diacetate (DCFH-DA) assay (Abcam Inc, Cambridge, MA, USA) to verify whether the initial 10 min AC stimulation leads to oxidative stress. The non-fluorescent substance DCFH-DA is incorporated by cells through the cell membrane. Inside the cell, the substance is deacetylated by esterases and cannot pass the cell membrane anymore. DCFH reacts with ROS like hydroxyl or peroxyl. Through the oxidation process 2, 7-dichlorofluorescein (DCF), which is highly fluorescent, is generated [34].

MG-63s were trypsinized, centrifuged, and washed with PBS. The cell pellet (1 × 10^6^ cells) was resuspended in DCFH-DA staining solution (20 µM) and incubated using ThermoMixer C (Eppendorf; 300 rpm at 37 °C in the dark) for 30 min. Afterward, the stained MG-63s were washed, centrifuged, and resuspended in phenol-red free DMEM (without pyruvate, Life technologies, Thermo Fisher Scientific) with 10% FCS and gentamycin. 40 µL of stained cells were seeded on coverslips in the 12-well plates, electrodes were placed on them, and AC stimulation started, or not at Control samples. After ES, 5% hydrogen peroxide (H_2_O_2_; Sigma-Aldrich, Milan, Italy) was pipetted in one Control sample as a positive check.

Immediately after stimulation, the DCF fluorescence was recorded by the plate reader infinite M200 (Tecan, Tecan i-control 1.9.17.0; Grödig, Austria) at an excitation of 485 nm and an emission of 535 nm. Subsequently, all samples were further cultivated in the incubator at 37 °C and removed at 0.5, 4, and 24 h for the ROS measurement. Four individual experiments with two respective technical replicates were done. The blank, i.e., the fluorescence intensity of the phenol-red free medium, was subtracted from the sample values.

### 2.11. Statistics

All in vitro experiments were repeated at least three times (unless otherwise stated) with independently passaged cells. The GraphPad Prism 7 software for Windows (GraphPad Software Inc., La Jolla, CA, USA) was used to evaluate statistical differences in in vitro experiments. The data are illustrated in a boxplot showing the median, the minima, and the maxima (median ± min/max). In the first step, we tested for normal distribution with the Shapiro–Wilk normality test (normal distribution: *p* > 0.05). Depending on the normal distribution, we used the parametric tests one-way ANOVA posthoc Bonferroni (paired values; medium characteristics, adhesion), one-way ANOVA posthoc uncorrected Fisher´s LSD (xCELLigence measurement, spreading, ROS production), or multiple *t*-tests (Ca^2+^ mobilization course).

For non-parametric procedures (no normal distribution), we applied the Friedman test (paired values) posthoc uncorrected Dunn’s test (Ca^2+^-level). We set the significance limit of *p* < 0.05 [35].

## 3. Results

### 3.1. Characterization of AC Electrical Stimulation (ES) Parameter

**Figure 3:**
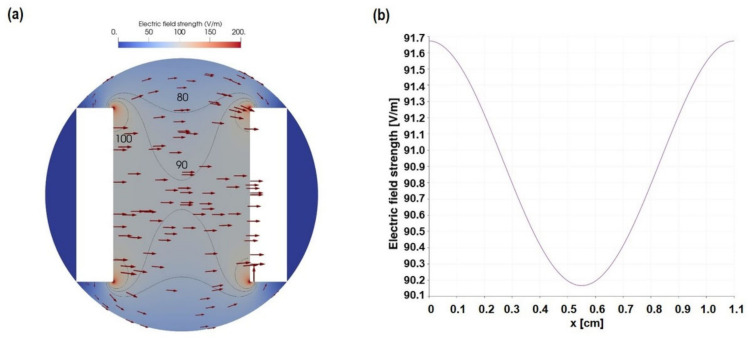
Simulation data revealed (**a**) the electric field distribution and strength (red arrows) between the two electrodes at a voltage difference of 1 V. The grey isolines highlight the regions where the field reaches 80, 90, and 100 V/m (the latter in the vicinity of the electrodes’ sharp corners), respectively. Furthermore, (**b**) shows the electric field strength along the centerline between the two electrodes. The result indicates that the cells are exerted to a mostly homogeneous field with a magnitude of about 90 V/m. Note that for different voltage differences, the field strength changes linearly w.r.t. the voltage.

The electric field strength and its magnitude between the electrodes can be seen in Figure 3. Between the electrodes, where the cells are placed, is a large region with a more or less homogeneous field. Only close to the electrode edges a field enhancement can be observed (Figure 3a). However, the area with an enhanced field is small in comparison to the area with a more or less constant field strength. Along the centerline of the well, the field decreases from 91.7 V/m at the electrodes to 90.2 V/m precisely at the midpoint between the electrodes (Figure 3b). Under the assumption of infinitely wide capacitor plates, the field could also be analytically estimated. In this case, it would be equal to the voltage difference over the electrode spacing. At a voltage difference of 1 V and a spacing of 1.1 cm, this corresponds to 90.91 V/m. It is of great interest to validate the numerical model. Since neither the electric field nor the electric potential in the well can be measured easily, the current through the electrode needs to be compared. The current equals the conductivity times the normal component of the electric field integrated over the electrode surface, yielding 1.75 A/m for the 2D geometry. With a 1 mL cell culture medium, a fill level of 3.4 mm is reached. It should be noted that capillary effects are not considered here. Then, the current is 5.95 mA for 1 S/m conductivity and scales linearly with the conductivity. Consequently, it would be 11.9 mA for 2 S/m and so on.

The individual simulated and measured electric parameters are summarized in Table 1. The measured currents are peak values during the pulse. See Chapter 4 for a comparative discussion of these values.

### 3.2. Acellular Medium Characteristics—AC-Stimulated Liquid

Analyzing the effect of AC stimulation on the culture medium is a crucial step in order to exclude changes in liquid properties, such as pH value, as a reason for the resulting cellular adaption/behavior.

**Figure 4:**
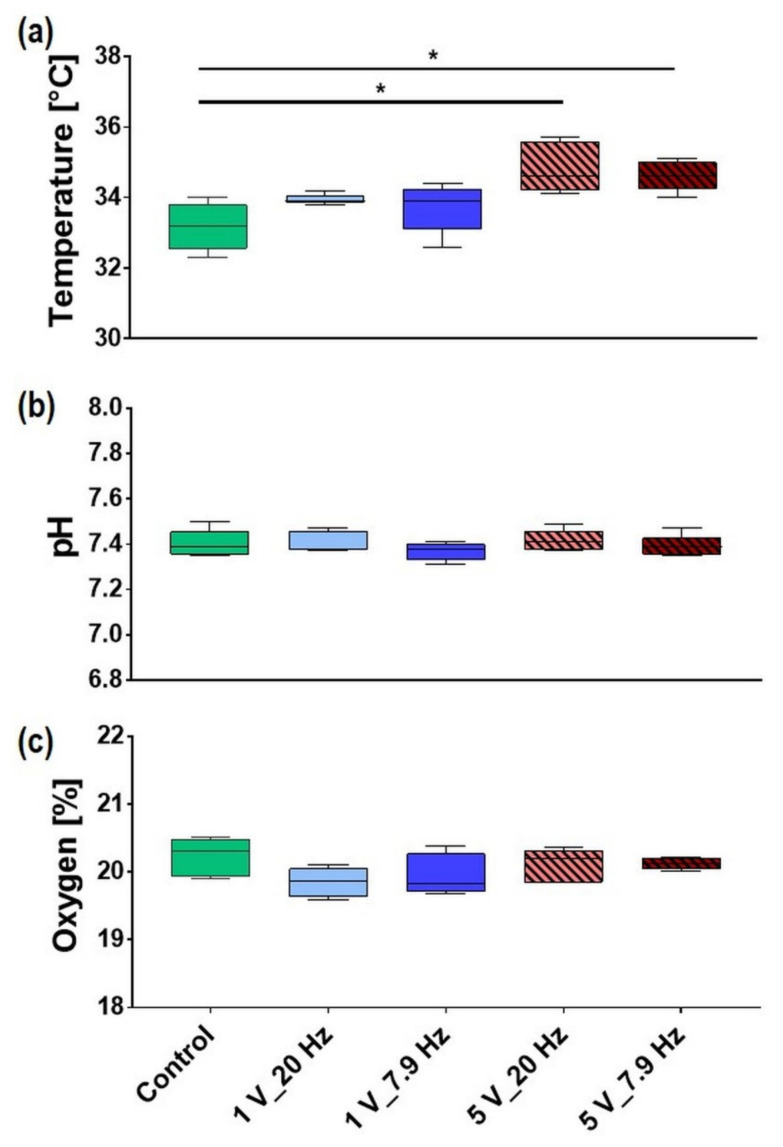
Properties of AC-stimulated liquids, i.e., the complete DMEM, after 10 min ES: (**a**) temperature, (**b**) pH, and (**c**) oxygen content. Note that only the temperature changed for the 5 V settings. (median ± min/max, *n* = 5–6, one-way ANOVA posthoc Bonferroni, * *p* < 0.05).

The temperature of the AC-stimulated medium was significantly changed vs. the Control only for the 5 V setups with 20 Hz and 7.9 Hz with 34.8 ± 0.3 °C and 34.6 ± 0.2 °C (mean ± s.e.m.), respectively. The mean value of the Control was 33.2 ± 0.3 °C (Figure 4a).

The pH values in the medium after ES did not change for 1 and 5 V in combination with 20 Hz and 7.9 Hz (e.g., Control pH 7.40 ± 0.03, 5 V_20 Hz pH 7.42 ± 0.02; mean ± s.e.m.) (Figure 4b).

The oxygen content in the medium was not changed after the 10 min AC stimulation (e.g., Control 20.2 ± 0.1%, 5 V_20 Hz 20.1 ± 0.09%; mean ± s.e.m.) (Figure 4c).

**Figure 5:**
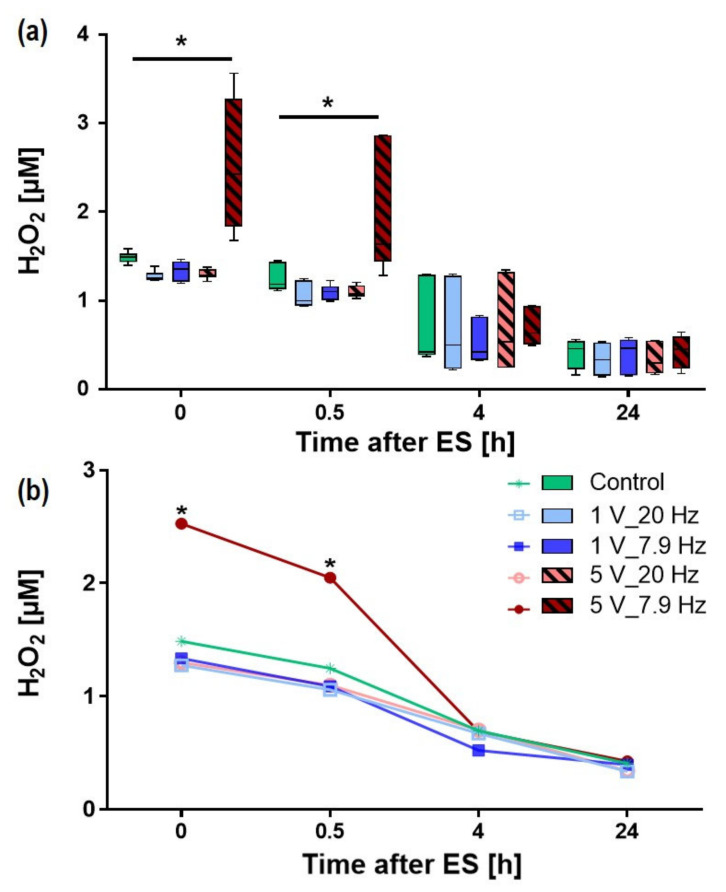
H_2_O_2_ content of AC-stimulated liquids, i.e., the DMEM w/o pyruvate, after 10 min ES, (**a**) for all AC-stimulation parameters as BoxPlot graph, (**b**) time course of H_2_O_2_ concentration. Note that 10 min AC with 1 V settings did not change the H_2_O_2_ concentration in contrast to 5 V_7.9 Hz with a significant increase in H_2_O_2_ up to 0.5 h. (Median ± min/max, *n* = 5–6, two-way ANOVA posthoc Bonferroni, * *p* < 0.05 compared to all).

During electrical stimulation, faradic byproducts such as H_2_O_2_ can be generated. H_2_O_2_ in the media can influence cellular fate and may contribute to ES-induced effects. In our experiments, the concentration of H_2_O_2_ was only increased in media stimulated with 5 V_7.9 Hz compared to the Control (Figure 5). The mean concentration increased from 1.48 µM ± 0.02 in Control samples to 2.52 µM ± 0.32 in 5 V_7.9 Hz samples directly after 10 min ES. The H_2_O_2_ concentration declined over time in all samples, with 5 V_ 7.9 Hz showing elevated levels also after 0.5 h (mean 2.05 ± 0.28 µM). After 4 h and 24 h, the concentration was similar in all samples and leveled off at about 0.38 µM (Figure 5a,b). The overall decline was independent of the O_2_ concentration in the media (data not shown).

### 3.3. AC-Stimulated Liquids and Long-Time Cell Adhesion

**Figure 6:**
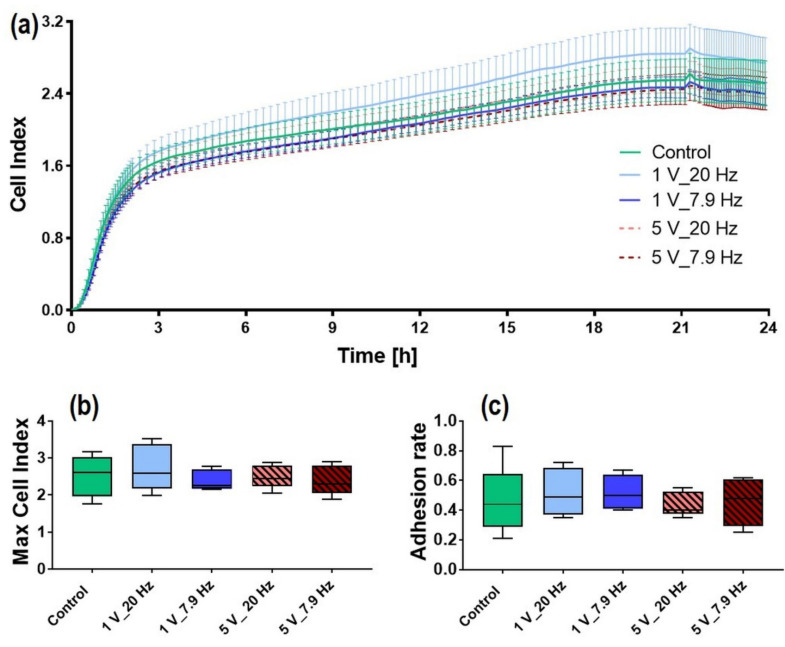
AC-stimulated liquid DMEM and influence on a long-time MG-63 cell adhesion via impedance measurement. (**a**) Cell adhesion and spreading expressed by the “Cell Index” parameter over 24 h. (xCELLigence RTCA; 5 independent experiments, mean ± s.e.m.; multiple t-tests, n.s.) (**b**) Maximal Cell Index (values after 24 h), and (**c**) corresponding cell adhesion rate (Cell Index at the time point when 50% of the cells adhere). Note that AC-stimulated liquids did not influence the cell’s adhesion capacity. (xCELLigence, 5 independent experiments; median ± min/max; one-way ANOVA posthoc uncorrected Fisher’s LSD, n.s.).

In order to analyze whether AC stimulation of liquids alone (rather than the cells) affected cell adhesion; we recorded the impedance with xCELLigence RTCA over 24 h. The adhering cells interfere with the electrode structures on the chip, thus impeding the electron response in the medium. The impedance depends on the number of cells, the cell size, and the strength of adhering cells. As shown in Figure 6, cells were not influenced by the AC-stimulated liquid, and the cell adhesion was approximately the same as the unstimulated Control. Solely a slight increase was detectable with 1 V_20 Hz AC-stimulated liquids (Figure 6a). After 24 h, the max cell index (i.e., the 24 h endpoint value) in MG-63s was comparable in all liquids indicating no changes of the liquid DMEM due to AC stimulation (Control: 2.5 ± 0.1, 1 V_20 Hz: 2.7 ± 0.1, 1 V_7.9 Hz: 2.4 ± 0.1, 5 V_20 Hz: 2.5 ± 0.1, 5 V_7.9 Hz: 2.4 ± 0.1; mean ± s.e.m.; Figure 6b). In addition, the adhesion rate (time point when 50% of the cells adhere) was the same in all samples and showed no influence of the AC-stimulated liquid (Control, 1 V_20 Hz, 1 V_7.9 Hz, 5 V_7.9 Hz: all 0.5; 5 V_20 Hz: 0.4; mean; Figure 6c).

### 3.4. AC-Stimulated Cells

#### 3.4.1. Initial Cell Adhesion

**Figure 7:**
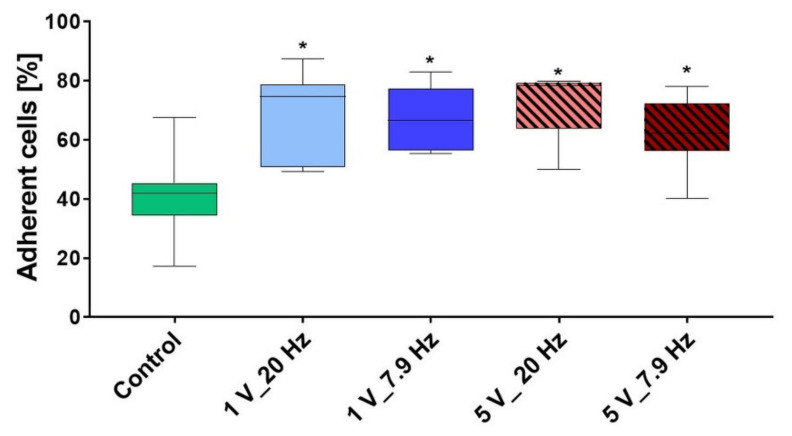
The adhesion of MG-63s after 10 min AC stimulation of the suspended cells. Note that after AC, the attachment of osteoblasts was significantly increased. (Cell count via FACSCalibur, median ± min/max, *n* = 7 independent experiments, one-way ANOVA posthoc Bonferroni, * *p* < 0.05 compared to Control).

In the initial phase of a few minutes, the cellular adhesion of suspended cells starts with cell attachment of still rounded cells on the sample surface. In our approach, flow cytometry was used to count the non-adhered cells, and then attached cells after 10 min AC stimulation were calculated in % of the adherent cells. In Figure 7, the influence of different electric field parameters on initial cell adhesion is shown. It is clearly to be seen, that the cells were activated, and adhesion (in %) was accelerated under AC (1 V_20 Hz: 67.7 ± 5.9, 1 V_7.9 Hz: 67.8 ± 3.9, 5 V_20 Hz: 71 ± 4.3, 5 V_7.9 Hz: 61.9 ± 4.6, Control: 40.9 ± 5.7; mean ± s.e.m.) (Figure 7). Interestingly, the activation of cell adhesion is independent of the field amplitude in the case of ES.

#### 3.4.2. Intracellular Calcium Ion (Ca^2+^) Level during AC stimulation

**Figure 8:**
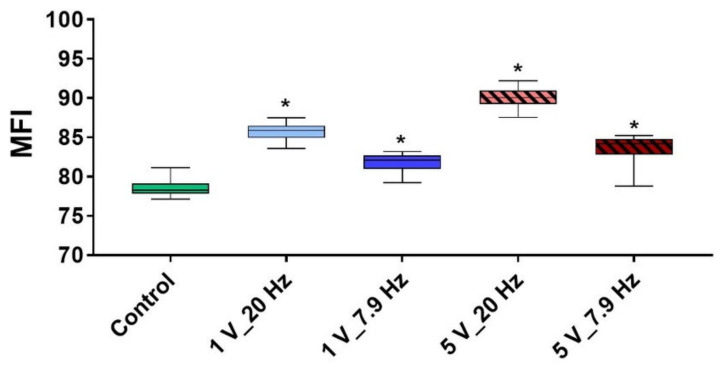
The level of intracellular calcium ions (Ca^2+^) in MG-63s within 10 min AC stimulation. Note that electrical stimulation activates the cells resulting in significantly higher Ca^2+^ levels in cells compared to the unstimulated Control. (LSM780, Carl Zeiss; time series of 30 cycles each 2 s started after 5 min settling phase of MG-63s; 7 independent experiments of defined areas of 10 cells, i.e., 70 cells per time point; median ± min/max; Friedman test posthoc uncorrected Dunn’s test, * *p* < 0.05 compared to Control).

The intracellular Ca^2+^ signaling has been suggested to play a critical role in the impact of electric fields on cells. Therefore, we first analyzed the immediate effect of AC stimulation on the Ca^2+^ level of fluo-3-stained MG-63s. The mean fluorescence intensity (MFI of Ca^2+^-signal) within 10 min AC stimulation of MG-63s is given in Figure 8. The AC electric field activated the cells, and the level of calcium ions increased significantly independent of the stimulation parameters used, but obviously with 20 Hz (1 V: 85.8 ± 0.2; 5 V: 90.1 ± 0.2; MFI mean ± s.e.m.) compared to the Control (78.5 ± 0.2; MFI mean ± s.e.m.).

#### 3.4.3. Calcium Ion Mobilization in AC-Pre-Stimulated Cells

**Figure 9:**
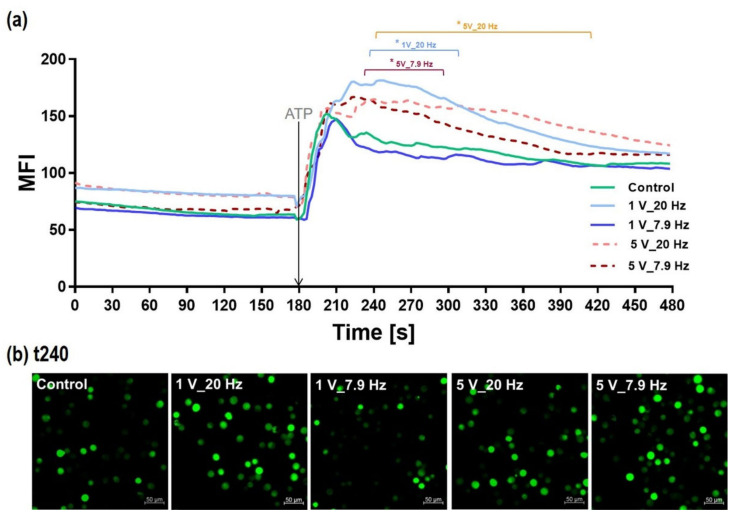
The intracellular calcium ion (Ca^2+^) signal of fluo-3-loaded MG-63s after 10 min electrical stimulation. (**a**) The time course of the Ca^2+^ signal (0–480 s) was immediately started after the 10 min AC stimulation (0 s). The adenosine triphosphate (ATP) addition was at time point 180 s. Note that electrical stimulation with 20 Hz (1 and 5 V) and 5 V_7.9 Hz pre-activates the cells resulting in a significantly increased mobilization of calcium ions after additional ATP. (Confocal microscope LSM780, Carl Zeiss; 6 independent experiments, defined areas of 10 cells per time point; polygon line as mean; multiple *t*-test, * *p* < 0.05 compared to Control). (**b**) Adequate fluorescence images of intracellular Ca^2+^ levels after ATP in electrically pre-stimulated cells (time point: 240 s). (LSM780, scale bars = 50 µm, 40× water objective).

It is obvious from Figure 8 that cells are activated during the 10 min AC stimulation. Figure 9 presents the time course of the intracellular Ca^2+^ signal of MG-63s immediately after ES. The basal intracellular Ca^2+^ ion level (time frame 0–179 s) is enhanced 1.2 times in cells that were pre-stimulated with the higher frequency of 20 Hz (Figure 9a). After an additional stimulus with the energy-rich molecule ATP at 180 s, the Ca^2+^ ions were mobilized significantly in these cells with a strong slope and a plateau phase (Figure 9a). The Ca^2+^ level after ATP increased significantly for AC pre-stimulated cells with the settings 5 V_20 Hz, 1 V_20 Hz, 5 V_7.9 Hz with MFI values of 164.9 ± 10.23, 178.2 ± 9.5, and 162.5 ± 9.9, respectively; compared to Controls (MFI: 131 ± 9.1), and 1 V_7.9 Hz (MFI: 119.5 ± 8.9) (Figure 9b). These experiments were carried out in a calcium-containing medium. To solve the question of whether ES directly mobilizes the Ca^2+^ ions from internal stores during the period 0–179 s or if we observe an influx of Ca^2+^ via the cell membrane, we used a calcium-free medium [36]. The additional experiments revealed that the basal Ca^2+^ level is also enhanced at least two times after ES (data not shown). Therefore, calcium ions are mobilized from intracellular stores.

#### 3.4.4. Cell Morphology

**Figure 10:**
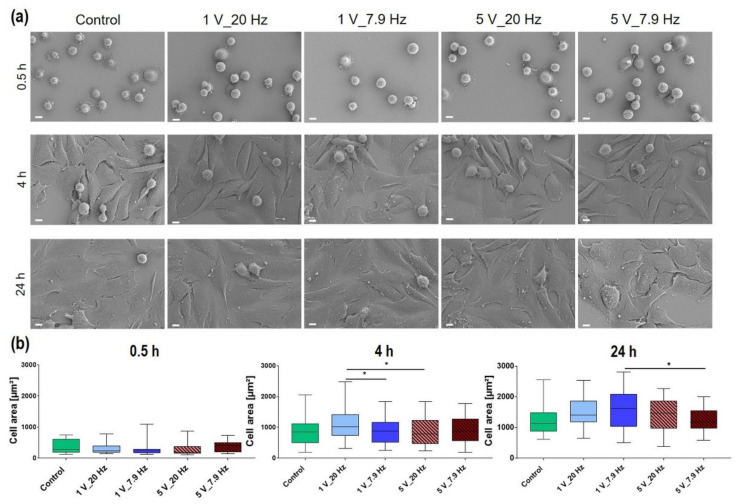
Influence of AC electrical stimulation (ES, 10 min) on MG-63 cell morphology and spreading at 0.5, 4, and 24 h. (**a**) Scanning electron microscopy of cells. Note that ES does not influence the overall cell shape. Cells start to spread at 0.5 h, which is typical for MG-63s. (FE-SEM Merlin compact, Carl Zeiss, 500×, scale bars = 10 µm). (**b**) Cell area analysis. Note that the different AC-stimulation settings have no significant impact on the time-dependent spreading compared to unstimulated control cells (Control). (ImageJ, *n* = 40 cells, median ± min/max; one-way ANOVA posthoc uncorrected Fisher’s LSD, * *p* < 0.05).

Physical factors can influence the cell morphology, e.g., physical plasma can shorten the microvilli on the cell membrane or inhibit the cell area [37]. Therefore, the influence of AC electrical stimulation on cell morphology and spreading behavior was observed. Cells were 10 min AC-stimulated, and then the cell areas were analyzed after 0.5, 4, and 24 h (Figure 10). With our AC stimulation using different parameter settings, we could not identify any changes in cell morphology compared to unstimulated cells (Control) (Figure 10a). The ES neither hampered nor significantly increased the spreading behavior (Figure 10b). A slight tendency of an increased cell area can be seen in 1 V_20 Hz cells after 4 and 24 h compared to Controls (Figure 10b). After 24 h the tendency of higher cell spreading can also be observed in 1 V_7.9 Hz stimulated cells (Table 2).

#### 3.4.5. Reactive Oxygen Species (ROS) Production

**Figure 11:**
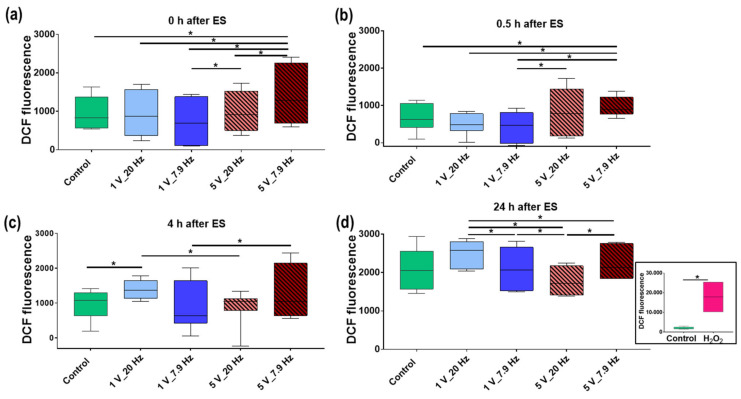
Reactive oxygen species (ROS) production in MG-63s after AC stimulation (10 min) over time (**a**) 0 h, (**b**) 0.5 h, (**c**) 4 h, and (**d**) 24 h. Note that 1 V settings did not change the intracellular ROS up to 0.5 h. In contrast, an increased ROS production could be observed after 5 V_7.9 Hz stimulation up to 0.5 h compared to Controls. At 4 h, a significant increase in ROS is then detectable in 1 V_20 Hz stimulated cells. However, after 24 h, all ROS levels were comparable to the Control. Note, the insert at 24 h plot (**d**) shows the maximum ROS production of cells treated with H_2_O_2_ (positive check, 5%). (DCF-DA test, Tecan, *n* = 4, median ± min/max; one-way ANOVA posthoc uncorrected Fisher’s LSD, * *p* < 0.05).

The stress level of MG-63s due to the AC stimulation was quantified using DCF-DA ROS detection assay over time: 0, 0.5, 4, and 24 h after ES. Figure 11a shows a significant increase of the ROS level directly after 5 V_7.9 Hz stimulation (mean fluorescence: 1412 ± 273) compared to the Control (mean fluorescence: 935 ± 151) as well as all other AC-stimulated MG-63s. This increased ROS production at 5 V_7.9 Hz was still significant after 0.5 h (mean fluorescence 0.5 h: 955 ± 92) (Figure 11b) but negligible after 4 h (median fluorescence 4 h: 1277 ± 267) compared to Control (mean fluorescence 4 h: 964 ± 147) (Figure 11c). An increase of one ROS species—H_2_O_2_—had also been detectable in the liquid stimulated with 5 V_7.9 Hz within the first minutes (see Section 3.2, Figure 5). After 4 h, however, the H_2_O_2_ concentrations in the media were approximately the same.

Nevertheless, from the 4 h time point, a significant increase in ROS production in MG-63s was detectable with 10 min of 1 V_20 Hz stimulation (mean fluorescence 4 h: 1381 ± 96) (Figure 11c). However, after 24 h, all ROS levels were comparable to the Control (mean fluorescence 24 h: 2029 ± 223), only among each other, a significant increase in ROS level in MG-63s under 1 V_20 Hz stimulation (mean fluorescence 24 h: 2496 ± 145) comparable to all other AC stimulations could be observed (Figure 11d). The fact that the overall intracellular ROS level is 2-fold higher after 24 h compared to the earlier time points can be explained by the doubling of cell numbers [38].

Increased production of intracellular ROS could be induced within 4 h in 5 V_7.9 Hz stimulated MG-63s and after 4 h in 1 V_ 20 Hz stimulated cells. Treatment with H_2_O_2_, as a positive check, resulted in a pronounced production of ROS (6.4-fold to Control after 24 h) and thus cellular stress, which finally led to the detachment of MG-63s (data not shown).

## 4. Discussion

Electrical stimulation (ES) plays a role in physiological processes in various tissues such as nerves, heart, cartilage, muscles [39], and bone [1,2,5,38]. Therefore, exogenous ES is a popular and successful adjunct therapy in vitro as well as in vivo for bone regeneration [39,40,41,42,43,44,45]. In the present study, we investigated the response of human osteoblasts (MG-63s) to 10 min ES by the multi-channel electrical stimulator of IonOptix (C-PACE EM) when seeding directly in the 12-well C-Dish chamber. The purpose of this study is threefold: (i) characterizing the electric fields; (ii) the influence of ES (AC) on liquid characteristics (DMEM); and (iii) influence of ES (AC) on the initial cell adhesion and calcium ion (Ca^2+^) signaling of MG-63s. ES generated by IonOptix is described in studies with, e.g., myoblasts [46], or osteoblasts [12,14]. Ercan et al. [12], showed a positive effect of IonOptix AC stimulation on osteoblast (hFOB 1.19) proliferation, but they technically modified the system with potentiometers to achieve a physiological current. In general, the comparability and interpretation of studies are very challenging due to the use of different systems (mostly homemade), type of ES, electrode configuration, experimental timeline, stimulation parameters, and hence the resulting electric fields, or the absence of essential data [5,24,40,47]. Budde et al. [24], published a study to draw attention to these misunderstandings and to explain which documentation is necessary for in vitro experiments, such as detailed information about electrical devices, electrode material, and experimental setups like waveform or exposure duration. Simulations could then be used to identify and explain parameters that affect the physiology of the cells [24]. However, the optimal ES parameters and the underlying mechanism are still unclear concerning the impact of cell behavior [5].

In the first part, we characterized the C-Dish chamber and the generated electric fields. The electric field is generated between two parallel graphite plates (i.e., a plate capacitor) 1.1 cm apart and in direct contact with the cells. We utilized a commercially available device from IonOptix (voltage controlled), which has been described earlier [12,14,46]. The IonOptix stimulator generates AC electric fields with constant biphasic rectangular pulses at a frequency of 7.9 or 20 Hz and voltage of 1 and 5 V, respectively.

The electric field (distribution and strength) depends on the electrode shape, its material, the surrounding media, and the type of ES. The simulation reveals a homogeneous field strength of 90 V/m at 1 V with our C-Dish chamber. The system can be assumed to be linear. Thus, the field should be about 450 V/m at 5 V. To prove this assumption, we measured the current at different voltages. Indeed, the current scales by about 5.6 when the voltage is increased by 5. Although this is not perfectly linear, it shows that the assumption of linearity is applicable.

The current through the medium can be considered to validate the simulation and hence the correctness of the estimated field [23]. We measured a current of 12 mA at 1V input amplitude. However, the simulations predicted a current of 7.7 mA using the measured conductivity of the cell culture medium (1.3 S/m). This deviation is unexpected but requires further analysis to identify possible reasons. For example, charging and discharging effects due to the capacitive electrode–electrolyte interface could change the shape of the current pulse. Possibilities to quantify the capacitive behavior are, for example, described in [23], but have not yet been implemented for the considered system. Other reasons could be measurement errors of the conductivity or the capillary effect in the dish, which has not been considered in the model.

Moreover, direct current (DC) can damage host tissues due to electrochemical reactions such as local pH increase [43], oxygen reduction, hydrogen peroxide (H_2_O_2_) formation, as well as the formation of free radicals and reactive oxygen species (ROS; such as hydrogen peroxide, hydroxyl) [5,15,44,48]. Electrochemical reactions such as corrosion phenomena due to the electrode material can also be prevented with graphene electrodes [15,42,49]. Furthermore, the use of AC, instead of DC, and further the pulsed ES prevents ion and protein accumulation near the electrodes [5,50], as well as the indirect effects like a pH increase [5,14,42,51].

For optimal growth of cells in vitro, we monitored pH, temperature, O_2−_, and H_2_O_2_ concentration of an acellular medium under ES in the present study. We could not detect any effects on pH and oxygen concentration in our AC-stimulated DMEM. Likewise, Ercan et al. [14], also found no significant change in the medium, both pH and temperature, after 1 h of ES (IonOptix, 1 V_20 Hz). However, we detected a slight temperature increase of 1.4 °C at 7.9 Hz and 1.6 °C at 20 Hz in the medium during AC stimulation at 5 V. This is consistent with a simple estimate based on the electric energy deposited into each well during stimulation (~10 J), particularly when considering thermal losses to the environment. For 1 V AC stimulation, the medium temperature increased continuously by about 0.6 °C every 5 min. Reissis et al. [52], demonstrated that mesenchymal stem cells (hMSCs) retain their morphology, metabolic activity, and viability when exposed to temperatures ranging up to 48 °C for at least 150 s. With our temperature increase up to 1.6 °C of the medium by the ES, we can assume no cell damage. Our in vitro results confirmed this assumption, as no morphological and growth impairment of MG-63s was detectable for all parameters after 10 min AC stimulation.

The H_2_O_2_ concentration in the medium increased significantly solely after stimulation with 5 V_7.9 Hz (2.52 µM). Srirussamee et al. [53], found H_2_O_2_ concentrations of acellular medium to be approximately 5 µM after two hours of DC stimulation. They applied the stimulated media to their cell culture, and the metabolic activity decreased in macrophages but not in MC3T3-E1 osteoblasts compared to the unstimulated Control. When ES was applied directly to the cells, all stimulated cells showed decreased metabolic activity [53]. An extracellular H_2_O_2_ concentration above 10 µM was described to lead to oxidative stress and cellular damage [54]. As we did not measure such high concentrations after ES, adverse effects through H_2_O_2_ are unlikely. Srirussamee et al. [53], suggested that the influence of other faradic byproducts in the media contributes to decreased metabolic activity after incubation with the stimulated medium. We hypothesized that the overall decline of H_2_O_2_ over 24 h may occur due to incubation at a 5% CO_2_ atmosphere in the incubator and consequently changed pH. The pH values of freshly opened medium and medium incubated for 24 h at standard culture conditions were unaltered. Only medium which had been open for several days at atmospheric CO_2_ pressure showed increased pH values which might correlate to increased H_2_O_2_ concentrations before incubation. The oxygen content in the media did not influence the H_2_O_2_ concentration (data not shown).

Similar to Srirussamee et al. [53], we applied the 10 min AC-stimulated acellular medium to our cell culture and found no effect on osteoblast adhesion, spreading, and growth within the first 24 h. We expected an effect because, e.g., another physical modification—a 60 s physical plasma treatment (Argon-plasma jet kINPen09) of the liquid DMEM—significantly affected cell vitality, microvilli, and tight junction formation [37]. Due to only minor changes in the temperature, but not for pH and oxygen content in the AC-stimulated medium, we can exclude this as a reason for the altered cell behavior. Balint et al. [43], also postulated that an influence of the surrounding medium on cell behavior can be excluded and that cells respond directly to the electric field. Electric fields are apparently sensed through the same pathways observed in mechanotransduction and chemotaxis [43].

Different cell types (like hMSC and osteoblasts) have been found to respond to various experimental ES conditions with altered cellular behaviors such as adhesion, proliferation, differentiation, and directed migration due to effects on signaling pathways [8,39,55]. A positive impact can only be achieved if the type of ES parameters such as intensity, frequency, and duration of electric field were well chosen [5,39,40,42]. Our ES parameters were chosen from the literature, where positive influence on osteoblasts has already been reported [12,14,56]. Ercan et al. [12], found that an optimal voltage window for maximizing osteoblast density on nanotubular titanium is 1 V to 15 V and 20 Hz (IonOptix). In the study of Aaron et al. [50], electric field amplitudes in the range of 10–1000 V/m were identified as sufficient to achieve an effect without cell damage; we are also within this range.

In our third part of the study, we demonstrated that cell adhesion was increased by 10 min ES, independent of AC-stimulation parameters. Enhanced cell attachment to surfaces due to ES has already been described in some studies [57,58,59]. Dubey et al. [60], showed that a 2 V pulsed ES strongly promoted the adhesion of L929 fibroblasts, whereas, at 25 V, the cells died. It is also described that, among others, ES influences the arrangement and density of adhesion receptors—integrins [41,61,62], or focal adhesion [63] such as vinculin [58,64]. In the subsequent studies, we also plan to examine integrin activation, such as ß1 integrin (9EG7), to confirm cell adhesion receptors’ involvement.

Cell attachment could influence cell behavior like cell signaling. We detected increased basal Ca^2+^ activity for settling cells under ES. However, the effect of ES on Ca^2+^ mobilization was dependent on frequencies and voltages: while at 1 V_7.9 Hz, reduced tendency was evident, all other ES parameters showed a significantly increased Ca^2+^ response after adenosine triphosphate (ATP) addition. Note that trypsinization of cells affects the calcium response after ATP addition [65], but we accept the systematic error in analyzing calcium signaling during and immediately after 10 min AC stimulation. A 10 min stimulation of 24 h adherent cells indicated no effect on calcium response, either basal or after ATP addition (data not shown). Many studies report that cells are sensitive to ES through activation of ion channels in the cell membrane, and release of Ca^2+^ from intracellular calcium stores like endoplasmic reticulum (ER) [8,13,15,39,42,43,45,57,66,67]. For example, Khatib et al. [68] described an increase in intracellular Ca^2+^ concentration in human osteoblasts after 20 min of DC stimulation at 2 V/cm due to an influx of extracellular Ca^2+^ through stretch-activated cation channels (SACC), a release from intracellular stores, as well as activation of phospholipase C (PLC). We primarily suspect an enhanced release of Ca^2+^ from intracellular stores, as we observe a strong response of cells by the addition of ATP, which activates the PLC pathway via G-protein coupled receptor, and finally releases Ca^2+^ from the ER [30]. Extracellular Ca^2+^ influx via ion channels is of secondary importance in our short 10 min stimulation, as analyses without extracellular Ca^2+^ sources from the HEPES buffer [36], also demonstrated an increased basal Ca^2+^ level in MG-63s in all ES parameters (data not shown). Zhou et al. [69], demonstrated that the intake of extracellular Ca^2+^ plays a decisive role in the increased calcium mobilization in MG-63s during nanosecond pulsed electric field stimulation.

In addition to Ca^2+^ signaling, many other cellular mechanisms, e.g., redox stress, are discussed to contribute to the effects of ES. Since direct ES stimulation at 5 V_7.9 Hz resulted in the generation of H_2_O_2_ in the acellular medium, we found the production of intracellular ROS in MG-63s within the first minutes to rise accordingly, which was then equalized to the level of the unstimulated Control after 4 h. Wu et al. [70] described a linear relationship between field strength and intracellular ROS production. However, the field strength alone could not be causative in our case, as an increase in intracellular ROS was detectable at 5 V_7.9 Hz but not at 5 V_20 Hz. However, from 4 h, there was an increase in intracellular ROS level at 1 V_20 Hz, suggesting that a signaling pathway was turned on by ES, like Ca^2+^ signaling. At 1 V_20 Hz, we also had the strongest Ca^2+^ release in response to ATP addition. Thus, Görlach et al. [21] postulated that Ca^2+^ and ROS signaling are inter-regulated and crucial for cell signaling. Diaz-Vegas et al. [71] found the production of intracellular ROS after ES in muscle fibers to be dependent on NOX2 activity. They found extracellular ATP to contribute to ROS production via P2Y1 receptors, also crosslinking Ca- and ROS-signaling. Studies indicated that intracellular ROS production could be increased by ES, acting as a signal transducer in physiological as well as pathological processes [15,57,72]. A 1.3-fold increase of ROS level in MG-63s at 1 V_20 Hz AC stimulation, compared to the Control (24 h), could be suggestive of a physiological level, according to our results, which triggers Ca^2+^ signaling. In contrast, the positive check (H_2_O_2_) showed a 6.4-fold increase in ROS production, leading to cell detachment and thus to a pathological process.

Sun et al. [67] demonstrated that alteration of ion gradients or cell surface charges by an applied electric field resulted in changes in Ca^2+^ signaling and subsequently increased gene expression of Col I & ALP but had no effect on hMSC growth rate or morphology. We also demonstrated the unaltered morphology of MG-63s under 10 min ES in FE-SEM images within 24 h. Furthermore, Sahm et al. [73] revealed in human primary osteoblast no altered proliferation and viability within 7 d AC stimulation on Ti6Al4V surfaces. Many studies describe improved cell survival and promote osseointegration (such synthesis of Collagen I) by ES [5,39,42,43,51,55,56,64,67]. Stimulation of osteoblasts with pulsed ES increased the calcium deposition after 3 weeks [14]. Furthermore, pulsed ES increased collagen production in pre-osteoblasts [5], demonstrating that pulsed stimulation can increase bone matrix deposition.

In this study, we have analyzed and validated a method for ES stimulation of osteoblasts in vitro based on direct contact of AC electric fields. The first results show that the cells are sensitive to AC stimulation and react with altered cell adhesion, redox status, and Ca^2+^ signaling, which are not due to changes in the liquid properties. In various studies, including ours, a positive effect on cell physiology was found using ES, through the activation of signaling pathways [42], such as Ca^2+^ or PI3K/Akt pathway [5,41,55,74]. Nevertheless, the duration of stimulation, as well as the parameters, need to be further optimized and automatically documented to allow the best possible adaptation. Thus, Pettersen et al. [5] reviewed short-term studies lasting between 2 h and 5 days.

In addition to the characterization and validation of the electrical stimulation system, the understanding of the molecular mechanisms of the electric fields is essential to optimize the stimulation parameters in bone healing.

## 5. Conclusions

Precisely characterizing the electric fields and their influence on the environment is crucial to comparing and evaluating in vitro stimulation studies. The liquid DMEM electrically stimulated with 1 V (7.9 and 20 Hz) did not change their pH, temperature, H_2_O_2_, and O_2_ content characteristics. Therefore, the AC-stimulated liquid alone did not influence the long-time MG-63 cell adhesion measured via impedance.

In contrast, the AC stimulation of suspended cells positively influences initial cell adhesion processes. Due to ES, the cells were more active, and a higher basal level of Ca^2+^ ions was detectable. Furthermore, an increase in voltage and pulse duration raises the Ca^2+^ mobilization and ROS production, which could trigger further signaling pathways.

Electrical stimulation has the potential for novel treatments in medical applications.

## Figures and Tables

**Table 1:** Individual ES values after measurement and simulation. To each setup, appropriate colors were dedicated (Code).

	Voltage [V]	Frequency [Hz]	Pulse Duration [ms]	Electric Field [V/m] *	Theoretical Electric Current [A] *	Measured Electric Current [A] **	Code
**Control**	-	-	-	-	-	-	
**1 V_20 Hz**	1 V	20	3.6	90	0.0077	0.012	
**1 V_7.9 Hz**	1 V	7.9	10	90	0.0077	0.012	
**5 V_20 Hz**	5 V	20	3.6	450	0.0385	0.067	
**5 V_7.9 Hz**	5 V	7.9	10	450	0.0385	0.069	

* Own calculation of electric field from simulation. ** Own measurement of electric current in DMEM with 10% FCS using Tektronix TDS2014B.

**Table 2:** Cell area values of 10 min AC-stimulated MG-63 osteoblastic cells (mean ± s.e.m.).

Time after AC Stimulation	Control (in µm^2^)	1 V_20 Hz (in µm^2^)	1 V_7.9 Hz (in µm^2^)	5 V_20 Hz (in µm^2^)	5 V_7.9 Hz (in µm^2^)
**0.5 h**	319 ± 5.1	295 ± 4.9	311.3 ± 10.0	322.6 ± 7.1	375.4 ± 5.7
**4 h**	903 ± 16.4	1075.9 ± 16.3	947.7 ± 10.9	890.6 ± 12.2	955.7 ± 13.1
**24 h**	1422.7 ± 14.2	1450.5 ± 17.4	1517.3 ± 23.2	1422.6 ± 23.7	1315.3 ± 11.5

## Data Availability

Not applicable.

## References

[1] Griffin M., Bayat A. (2011). Electrical Stimulation in Bone Healing: Critical Analysis by Evaluating Levels of Evidence. Eplasty.

[2] Fukada E., Yasuda I. (1957). On the Piezoelectric Effect of Bone. J. Phys. Soc. Jpn..

[3] Ghiasi M.S., Chen J., Vaziri A., Rodriguez E.K., Nazarian A. (2017). Bone fracture healing in mechanobiological modeling: A review of principles and methods. Bone Rep..

[4] Bassett C.A., Becker R.O. (1962). Generation of Electric Potentials by Bone in Response to Mechanical Stress. Science.

[5] Pettersen E., Anderson J., Ortiz-Catalan M. (2022). Electrical stimulation to promote osseointegration of bone anchoring implants: A topical review. J. Neuro Eng. Rehabil..

[6] O’Hearn S.F., Ackerman B.J., Mower M.M. (2016). Paced monophasic and biphasic waveforms alter transmembrane potentials and metabolism of human fibroblasts. Biochem. Biophys. Rep..

[7] Khitrin A.J., Khitrin K.A., Model M.A. (2014). A model for membrane potential and intracellular ion distribution. Chem. Phys. Lipids.

[8] Love M.R., Palee S., Chattipakorn S.C., Chattipakorn N. (2018). Effects of electrical stimulation on cell proliferation and apoptosis. J. Cell. Physiol..

[9] Pangalos M., Bintig W., Schlingmann B., Feyerabend F., Witte F., Begandt D., Heisterkamp A., Ngezahayo A. (2011). Action potentials in primary osteoblasts and in the MG-63 osteoblast-like cell line. J. Bioenerg. Biomembr..

[10] Rebl H., Finke B., Schmidt J., Mohamad H.S., Ihrke R., Helm C.A., Nebe J.B. (2016). Accelerated cell-surface interlocking on plasma polymer-modified porous ceramics. Mater. Sci. Eng. C Mater Biol. Appl..

[11] Kirson E.D., Dbalý V., Tovaryš F., Vymazal J., Soustiel J.F., Itzhaki A., Mordechovich D., Steinberg-Shapira S., Gurvich Z., Schneiderman R. (2007). Alternating electric fields arrest cell proliferation in animal tumor models and human brain tumors. Proc. Natl. Acad. Sci. USA.

[12] Ercan B., Webster T.J. (2008). Greater osteoblast proliferation on anodized nanotubular titanium upon electrical stimulation. Int. J. Nanomed..

[13] Bhavsar M.B., Cato G., Hauschild A., Leppik L., Oliveira K.M., Eischen-Loges M.J., Barker J.H. (2019). Membrane potential (V_mem_) measurements during mesenchymal stem cell (MSC) proliferation and osteogenic differentiation. PeerJ.

[14] Ercan B., Webster T.J. (2010). The effect of biphasic electrical stimulation on osteoblast function at anodized nanotubular titanium surfaces. Biomaterials.

[15] Kloth L.C. (2014). Electrical Stimulation Technologies for Wound Healing. Adv. Wound Care.

[16] Thrivikraman G., Boda S.K., Basu B. (2018). Unraveling the mechanistic effects of electric field stimulation towards directing stem cell fate and function: A tissue engineering perspective. Biomaterials.

[17] Manolagas S.C., Almeida M. (2007). Gone with the Wnts: β-Catenin, T-Cell Factor, Forkhead Box O, and Oxidative Stress in Age-Dependent Diseases of Bone, Lipid, and Glucose Metabolism. Mol. Endocrinol..

[18] Thannickal V.J., Fanburg B.L. (2000). Reactive oxygen species in cell signaling. Am. J. Physiol. Lung Cell. Mol. Physiol..

[19] Dröge W. (2002). Free Radicals in the Physiological Control of Cell Function. Physiol. Rev..

[20] Verma N., Pink M., Schmitz-Spanke S. (2021). A new perspective on calmodulin-regulated calcium and ROS homeostasis upon carbon black nanoparticle exposure. Arch. Toxicol..

[21] Görlach A., Bertram K., Hudecova S., Krizanova O. (2015). Calcium and ROS: A mutual interplay. Redox Biol..

[22] Tandon N., Cannizzaro C., Figallo E., Voldman J., Vunjak-Novakovic G. (2006). Characterization of Electrical Stimulation Electrodes for Cardiac Tissue Engineering. Conf. Proc. IEEE Eng. Med. Biol. Soc..

[23] Zimmermann J., Budde K., Arbeiter N., Molina F., Storch A., Uhrmacher A.M., van Rienen U. (2021). Using a Digital Twin of an Electrical Stimulation Device to Monitor and Control the Electrical Stimulation of Cells in vitro. Front. Bioeng. Biotechnol..

[24] Budde K., Zimmermann J., Neuhaus E., Schroder M., Uhrmacher A.M., van Rienen U. (2019). Requirements for Documenting Electrical Cell Stimulation Experiments for Replicability and Numerical Modeling. Annu. Int. Conf. IEEE Eng. Med. Biol. Soc..

[25] Staehlke S., Rebl H., Nebe B. (2019). Phenotypic stability of the human MG-63 osteoblastic cell line at different passages. Cell Biol. Int..

[26] Czekanska E.M., Stoddart M.J., Ralphs J.R., Richards R.G., Hayes J.S. (2014). A phenotypic comparison of osteoblast cell lines versus human primary osteoblasts for biomaterials testing. J. Biomed. Mater. Res. Part A.

[27] Pautke C., Schieker M., Tischer T., Kolk A., Neth P., Mutschler W., Milz S. (2004). Characterization of osteosarcoma cell lines MG-63, Saos-2 and U-2 OS in comparison to human osteoblasts. Anticancer Res..

[28] Artun F.T., Karagöz A. (2021). Antiproliferative and apoptosis inducing effects of the methanolic extract of Centaurea hermannii in human cervical cancer cell line. Biotech. Histochem..

[29] Bergemann C., Waldner A.-C., Emmert S., Nebe J.B. (2020). The Hyaluronan Pericellular Coat and Cold Atmospheric Plasma Treatment of Cells. Appl. Sci..

[30] Staehlke S., Oster P., Seemann S., Kruse F., Brief J., Nebe B. (2022). Laser Structured Dental Zirconium for Soft Tissue Cell Occupation—Importance of Wettability Modulation. Materials.

[31] Staehlke S., Koertge A., Nebe B. (2015). Intracellular calcium dynamics dependent on defined microtopographical features of titanium. Biomaterials.

[32] Schröder M., Staehlke S., Groth P., Nebe J.B., Spors S., Krüger F. (2022). Structure-based knowledge acquisition from electronic lab notebooks for research data provenance documentation. J. Biomed. Semant..

[33] Staehlke S., Nebe J.B. (2021). Research data of Calcium Imaging after electrical stimulation [Data set]. Zenodo.

[34] Chen X., Zhong Z., Xu Z., Chen L., Wang Y. (2010). 2′,7′-Dichlorodihydrofluorescein as a fluorescent probe for reactive oxygen species measurement: Forty years of application and controversy. Free Radic. Res..

[35] Dunn O.J. (1964). Multiple Comparisons Using Rank Sums. Technometrics.

[36] Gruening M., Neuber S., Nestler P., Lehnfeld J., Dubs M., Fricke K., Schnabelrauch M., Helm C.A., Müller R., Staehlke S. (2020). Enhancement of Intracellular Calcium Ion Mobilization by Moderately but Not Highly Positive Material Surface Charges. Front. Bioeng. Biotechnol..

[37] Hoentsch M., Bussiahn R., Rebl H., Bergemann C., Eggert M., Frank M., von Woedtke T., Nebe B. (2014). Persistent Effectivity of Gas Plasma-Treated, Long Time-Stored Liquid on Epithelial Cell Adhesion Capacity and Membrane Morphology. PLoS ONE.

[38] Clover J., Gowen M. (1995). Are MG-63 and HOS TE85 human osteosarcoma cell lines representative models of the osteoblastic phenotype?. Bone.

[39] Jin G., Yang G.-H., Kim G. (2014). Tissue engineering bioreactor systems for applying physical and electrical stimulations to cells. J. Biomed. Mater. Res. Part B Appl. Biomater..

[40] Nicksic P.J., Donnelly D.T., Hesse M., Bedi S., Verma N., Seitz A.J., Shoffstall A.J., Ludwig K.A., Dingle A.M., Poore S.O. (2022). Electronic Bone Growth Stimulators for Augmentation of Osteogenesis in In Vitro and In Vivo Models: A Narrative Review of Electrical Stimulation Mechanisms and Device Specifications. Front. Bioeng. Biotechnol..

[41] Aleem I.S., Aleem I., Evaniew N., Busse J.W., Yaszemski M., Agarwal A., Einhorn T., Bhandari M. (2016). Efficacy of Electrical Stimulators for Bone Healing: A Meta-Analysis of Randomized Sham-Controlled Trials. Sci. Rep..

[42] Chen C., Bai X., Ding Y., Lee I.-S. (2019). Electrical stimulation as a novel tool for regulating cell behavior in tissue engineering. Biomater. Res..

[43] Balint R., Cassidy N.J., Cartmell S.H. (2013). Electrical Stimulation: A Novel Tool for Tissue Engineering. Tissue Eng. Part B Rev..

[44] Haglin J.M., Jain S., Eltorai A.E., Daniels A.H. (2017). Bone Growth Stimulation: A critical analysis review. JBJS Rev..

[45] Brighton C.T., Wang W., Seldes R., Zhang G.H., Pollack S.R. (2001). Signal Transduction in Electrically Stimulated Bone Cells. J. Bone Jt. Surg..

[46] Bydak B., Pierdoná T.M., Seif S., Sidhom K., Obi P.O., Labouta H.I., Gordon J.W., Saleem A. (2022). Characterizing Extracellular Vesicles and Particles Derived from Skeletal Muscle Myoblasts and Myotubes and the Effect of Acute Contractile Activity. Membranes.

[47] Taghian T., Narmoneva D.A., Kogan A.B. (2015). Modulation of cell function by electric field: A high-resolution analysis. J. R. Soc. Interface.

[48] Asadi M.R., Torkaman G. (2014). Bacterial Inhibition by Electrical Stimulation. Adv. Wound Care.

[49] Contu F., Elsener B., Böhni H. (2002). Characterization of implant materials in fetal bovine serum and sodium sulfate by electrochemical impedance spectroscopy. I. Mechanically polished samples. J. Biomed. Mater. Res..

[50] Aaron R.K., Ciombor D.M., Wang S., Simon B. (2006). Clinical Biophysics: The Promotion of Skeletal Repair by Physical Forces. Ann. N. Y. Acad. Sci..

[51] Dauben T.J., Ziebart J., Bender T., Zaatreh S., Kreikemeyer B., Bader R. (2016). A Novel In Vitro System for Comparative Analyses of Bone Cells and Bacteria under Electrical Stimulation. BioMed Res. Int..

[52] Reissis Y., García-Gareta E., Korda M., Blunn G.W., Hua J. (2013). The effect of temperature on the viability of human mesenchymal stem cells. Stem Cell Res. Ther..

[53] Srirussamee K., Mobini S., Cassidy N.J., Cartmell S.H. (2019). Direct electrical stimulation enhances osteogenesis by inducing Bmp2 and Spp1 expressions from macrophages and preosteoblasts. Biotechnol. Bioeng..

[54] Sies H. (2017). Hydrogen peroxide as a central redox signaling molecule in physiological oxidative stress: Oxidative eustress. Redox Biol..

[55] Meng S., Rouabhia M., Zhang Z., Gargiulo G.D., McEwan A. (2011). Electrical Stimulation in Tissue Regeneration. Applied Biomedical Engineering.

[56] Stephan M., Zimmermann J., Klinder A., Sahm F., Van Rienen U., Kämmerer P.W., Bader R., Jonitz-Heincke A. (2020). Establishment and Evaluation of an In Vitro System for Biophysical Stimulation of Human Osteoblasts. Cells.

[57] Leppik L., Oliveira K.M.C., Bhavsar M.B., Barker J.H. (2020). Electrical stimulation in bone tissue engineering treatments. Eur. J. Trauma Emerg. Surg..

[58] Bodhak S., Bose S., Kinsel W.C., Bandyopadhyay A. (2012). Investigation of in vitro bone cell adhesion and proliferation on Ti using direct current stimulation. Mater. Sci. Eng. C Mater. Biol. Appl..

[59] Li Y., Li X., Zhao R., Wang C., Qiu F., Sun B., Ji H., Qiu J., Wang C. (2017). Enhanced adhesion and proliferation of human umbilical vein endothelial cells on conductive PANI-PCL fiber scaffold by electrical stimulation. Mater. Sci. Eng. C.

[60] Dubey A.K., Gupta S.D., Basu B. (2011). Optimization of electrical stimulation parameters for enhanced cell proliferation on biomaterial surfaces. J. Biomed. Mater. Res. Part B Appl. Biomater..

[61] Sjaastad M.D., Nelson W.J. (1997). Integrin-mediated calcium signaling and regulation of cell adhesion by intracellular calcium. BioEssays.

[62] Sun S., Titushkin I., Cho M. (2006). Regulation of mesenchymal stem cell adhesion and orientation in 3D collagen scaffold by electrical stimulus. Bioelectrochemistry.

[63] Katoh K. (2022). Effects of Electrical Stimulation on the Signal Transduction-Related Proteins, c-Src and Focal Adhesion Kinase, in Fibroblasts. Life.

[64] Kumar A., Nune K.C., Misra R. (2016). Understanding the response of pulsed electric field on osteoblast functions in three-dimensional mesh structures. J. Biomater. Appl..

[65] Strong A.D., Daniels R.L. (2017). Live-cell calcium imaging of adherent and non-adherent GL261 cells reveals phenotype-dependent differences in drug responses. BMC Cancer.

[66] Massari L., Benazzo F., Falez F., Perugia D., Pietrogrande L., Setti S., Osti R., Vaienti E., Ruosi C., Cadossi R. (2019). Biophysical stimulation of bone and cartilage: State of the art and future perspectives. Int. Orthop..

[67] Sun S., Liu Y., Lipsky S., Cho M. (2007). Physical manipulation of calcium oscillations facilitates osteodifferentiation of human mesenchymal stem cells. FASEB J..

[68] Khatib L., Golan D.E., Cho M. (2004). Physiologic electrical stimulation provokes intracellular calcium increase mediated by phospholipase C activation in human osteoblasts. FASEB J..

[69] Zhou P., He F., Han Y., Liu B., Wei S. (2018). Nanosecond pulsed electric field induces calcium mobilization in osteoblasts. Bioelectrochemistry.

[70] Wu S.-Y., Hou H.-S., Sun Y.-S., Cheng J.-Y., Lo K.-Y. (2015). Correlation between cell migration and reactive oxygen species under electric field stimulation. Biomicrofluidics.

[71] Díaz-Vegas A., Campos C.A., Contreras-Ferrat A., Casas M., Buvinic S., Jaimovich E., Espinosa A. (2015). ROS Production via P2Y1-PKC-NOX2 Is Triggered by Extracellular ATP after Electrical Stimulation of Skeletal Muscle Cells. PLoS ONE.

[72] Snezhkina A.V., Kudryavtseva A.V., Kardymon O.L., Savvateeva M.V., Melnikova N.V., Krasnov G.S., Dmitriev A.A. (2019). ROS Generation and Antioxidant Defense Systems in Normal and Malignant Cells. Oxidative Med. Cell. Longev..

[73] Sahm F., Ziebart J., Jonitz-Heincke A., Hansmann D., Dauben T., Bader R. (2020). Alternating Electric Fields Modify the Function of Human Osteoblasts Growing on and in the Surroundings of Titanium Electrodes. Int. J. Mol. Sci..

[74] Bagne L., Oliveira M.A., Pereira A.T., Caetano G.F., Oliveira C.A., Aro A.A., Chiarotto G.B., Santos G.M.T., Mendonça F.A.S., Santamaria-Jr M. (2021). Electrical therapies act on the Ca^2+^/CaM signaling pathway to enhance bone regeneration with bioactive glass [S53P4] and allogeneic grafts. J. Biomed. Mater. Res. Part B Appl. Biomater..

